# Kidney concentrating capacity in children with autosomal recessive polycystic kidney disease is linked to glomerular filtration and hypertension

**DOI:** 10.1007/s00467-022-05834-5

**Published:** 2022-12-20

**Authors:** Tomáš Seeman, Kveta Bláhová, Filip Fencl, Richard Klaus, Bärbel Lange-Sperandio, Gabriela Hrčková, Ĺudmila Podracká

**Affiliations:** 1 Department of Pediatrics, Dr. v. Hauner Children’s Hospital, University Hospital, Ludwig Maximilian University Munich, Lindwurmstraße 4, 80337 Munich, Germany; 2 Department of Pediatrics, 2nd Medical Faculty, Charles University, Prague, Czech Republic; 3 Department of Pediatrics, University Hospital Ostrava, Ostrava, Czech Republic; 4 Department of Pediatrics, Medical Faculty, Comenius University and National Institute of Children’s Diseases, Bratislava, Slovakia

**Keywords:** Glomerular filtration rate, Hypertension, Kidney length, Pediatric population

## Abstract

**Background:**

Impaired kidney concentration capacity is present in half of the patients with autosomal dominant polycystic kidney disease (ADPKD). The kidney concentrating capacity was further impaired within the animal model of autosomal recessive polycystic kidney disease (ARPKD). To date, only one small study has investigated it in children having ARPKD. Therefore, we aimed to study the kidney concentrating ability in a larger cohort of children with ARPKD.

**Methods:**

Eighteen children (median age 8.5 years, range 1.3–16.8) were retrospectively investigated. A standardized kidney concentrating capacity test was performed after the application of a nasal drop of desmopressin (urine osmolality > 900 mOsmol/kg). The glomerular filtration rate was estimated using the Schwartz formula (eGFR) and blood pressure (BP) was measured as office BP.

**Results:**

Kidney concentrating capacity was decreased (urine osmolality < 900 mOsmol/kg) in 100% of children with ARPKD. The median urine osmolality after desmopressin application was 389 (range 235–601) mOsmol/kg. Sixteen patients (89%) were defined as hypertensive based on their actual BP level or their use of antihypertensive drugs. The maximum amounts of urinary concentration correlated significantly with eGFR (*r* = 0.72, *p* < 0.0001) and hypertensive scores (*r* = 0.50, *p* < 0.05), but not with kidney size. Twelve patients (67%) were defined as having CKD stages 2–4. The median concentrating capacity was significantly lower in children within this group, when compared to children with CKD stage 1 possessing a normal eGFR (544 mOsmol/kg, range 413–600 mOsmol/kg vs. 327 mOsmol/kg, range 235–417 mOsmol/l, *p* < 0.001).

**Conclusions:**

Impaired kidney concentrating capacity is present in most children with ARPKD and is associated with decreased eGFR and hypertension.

**Graphical abstract:**

**Figure:**
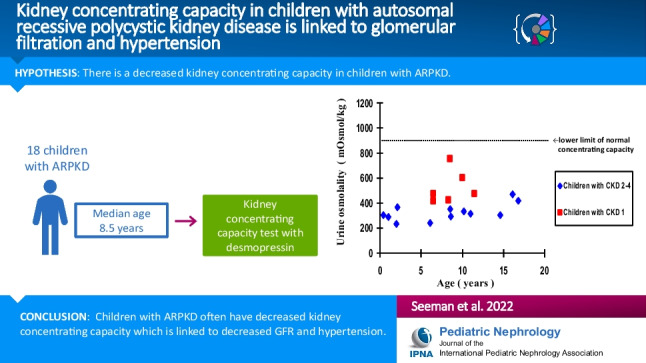
A higher resolution version of the Graphical abstract is available as  Supplementary information

**Supplementary information:**

The online version contains supplementary material available at 10.1007/s00467-022-05834-5.

## Introduction

Autosomal recessive polycystic kidney disease (ARPKD) is a rare but severe disease with a high mortality rate and increased morbidity leading to childhood chronic kidney failure in approximately 50% of children [1, 2]. It is primarily a tubular disorder, and it affects mainly children, but late manifestations in adulthood also exist [3, 4]. Kidney concentrating ability test is a simple measure of the function of the collecting duct and distal tubule, which has been demonstrated to be impaired in about half of adults, as well as pediatric patients with autosomal dominant polycystic kidney disease (ADPKD) [5–8]. However, there has been only one small study on the concentrating capacity in patients with ARPKD from 1975, which was performed on five children [9]. There is also a finding from an experimental study on a rat model of ARPKD that showed impaired urine concentrating capacity in these animals [10].

We aimed to study kidney concentrating capacity in a larger cohort of children with ARPKD and to elucidate the possible association between concentrating capacity, glomerular filtration rate, blood pressure (BP), hypertension, and kidney size.

## Patients and methods

Forty-nine patients with ARPKD were managed from 1996 to 2020 within our three tertiary pediatric kidney clinics in Prague, Bratislava, and Munich. We have retrospectively identified from medical records 18 patients with ARPKD who had urine concentrating capacity performed (*n* = 14/3/1 from Prague/Bratislava/Munich). The demographic and clinical data, antihypertensive medication, symptoms, and complications of children with ARPKD are shown in Table 1. Thirteen patients were described in our earlier paper on ambulatory blood pressure in ARPKD [11].

**Table 1:** Characteristics of the patients with ARPKD. Data are medians and interquartile range (IQR)

Age at the study (years)	8.5 (2.4–14.7)
Sex (males:females)	10:8
Age at manifestation (years)	0.7 (0.0–6.7)
*PKHD1*−gene variant (homozygous or compound heterozygous)	11 (in the remaining 7 patients DNA analysis not done)
Antihypertensive medication	*n* = 13 (72%)
Mean kidney length of both kidneys (range)	4.8 SDS (0.6–8.5)

The diagnosis of ARPKD was established using phenotype characteristics (typical kidney ultrasound with enlarged hyperechogenic kidneys with poor corticomedullary differentiation with/without small cysts) and characteristic hepatic involvement with liver fibrosis). These criteria were completed together with normal kidney ultrasounds from both parents, consistent with autosomal-recessive inheritance of kidney disease [2]. Moreover, in eleven of these eighteen patients, pathogenic homozygous or compound heterozygous mutations of the *PKHD1* gene were detected. In the remaining seven patients, genetic analysis was not done.

### Kidney concentrating capacity

We performed a morning standardized kidney concentrating ability test according to Janda et al.; urine osmolality was measured four hours after intranasal application of 5 μg/5 kg body weight 1-deamino-8-D-arginine-vasopressin – desmopressin (DDAVP, Ferring, CZE) [12]. Any amount of fluid intake was disallowed for 4 h and urine osmolality was measured after the application of desmopressin in the urine collected during this 4-h period (Roeblig osmometer was used and urine osmolality expressed in mOsmol/kg). There were no children who suffered from rhinitis or acute intercurrent illness at the time we performed the kidney concentrating test and no complications were observed while performing the investigation. Impaired kidney concentrating ability was defined as urine osmolality < 900 mOsmol/kg [12].

### Glomerular filtration

Serum creatinine was measured on the same day as the administration of desmopressin (Jaffé or enzymatic)**.** The glomerular filtration rate (GFR) was estimated by Schwartz formula [13, 14]. Chronic kidney disease (CKD) stages were classified according to Kidney Disease Improving Global Outcomes recommendations [15].

### *Blood pressure*

Office blood pressure (BP) was measured on the same day as the administration of desmopressin using a conventional mercury manometer, while hypertension on the other hand was diagnosed as systolic and/or diastolic BP ≥ 95th percentile, according to appropriate recent European recommendations [16],or taking antihypertensives. Sixteen children received antihypertensives: 14 angiotensin−converting enzyme inhibitors, 1 angiotensin receptor blocker, 9 beta−blockers, 9 calcium channel blockers, and 1 diuretic. The office blood pressure index (BPI) was calculated as BP divided by the 95th percentile. The heaviness of hypertension (HT) was staged by a modified hypertension score according to Guidi et al. [17]^.^ The hypertension scores were calculated for every child; each antihypertensive = one point and hypertension discovered through office BP measurements, regardless of drugs administered = 1 point.

### *Kidney ultrasound*

A kidney ultrasound was performed at the same time, or up to 6 months before or after the desmopressin test (5−MHz transducers: either an Acuson 128 PX−10 Siemens Medical Solutions, Erlangen, Germany or a 270 SSA: Toshiba Medical Systems, Tokyo, Japan). The size of the kidney (mm) was expressed as standard deviation score (SDS) [18].

### Ethical considerations

The present trial was conducted in accordance with the principles outlined in the Declaration of Helsinki. In view of the retrospective nature of the study, ethical approval was waived by the local ethics committee at the University Hospital Motol in Prague and Bratislava and all procedures performed were part of the routine care of patients with ARPKD.

### Statistical analysis

As the variables were irregularly distributed, all continuous variables were expressed using medians and interquartile ranges; Wilcoxon test was used for univariate comparison. The Spearman rank correlation test was used to assess correlations between continuous variables. Multivariate regression analysis was performed using urine osmolality as an outcome variable along with antihypertensive scores, with blood pressure and eGFR as predictor variables. All statistical tests were done using R statistical software; *p* < 0.05 was regarded to be statistically significant.

## Results

**Fig. 1:**
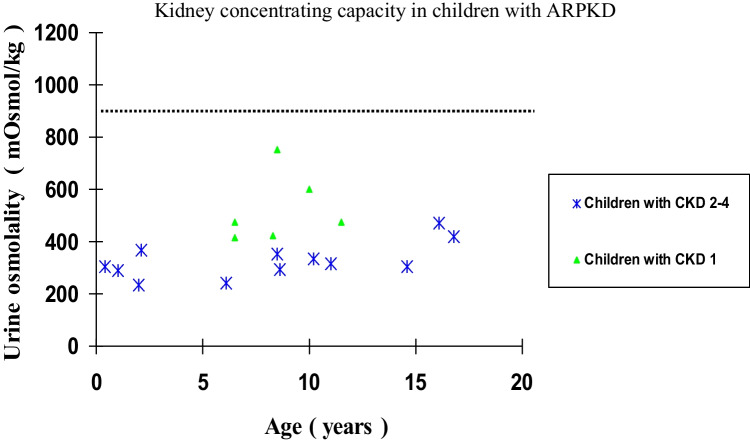
Kidney concentrating capacity in children with ARPKD (dotted line represents the lower limit of normal concentrating capacity)

Kidney concentrating ability was shown to be impaired in all 18 children (100%). Median urine osmolality was 360 mOsmol/kg (IQR 302–459). Data of urine osmolality of individual patients are given in Fig. 1.

**Fig. 2:**
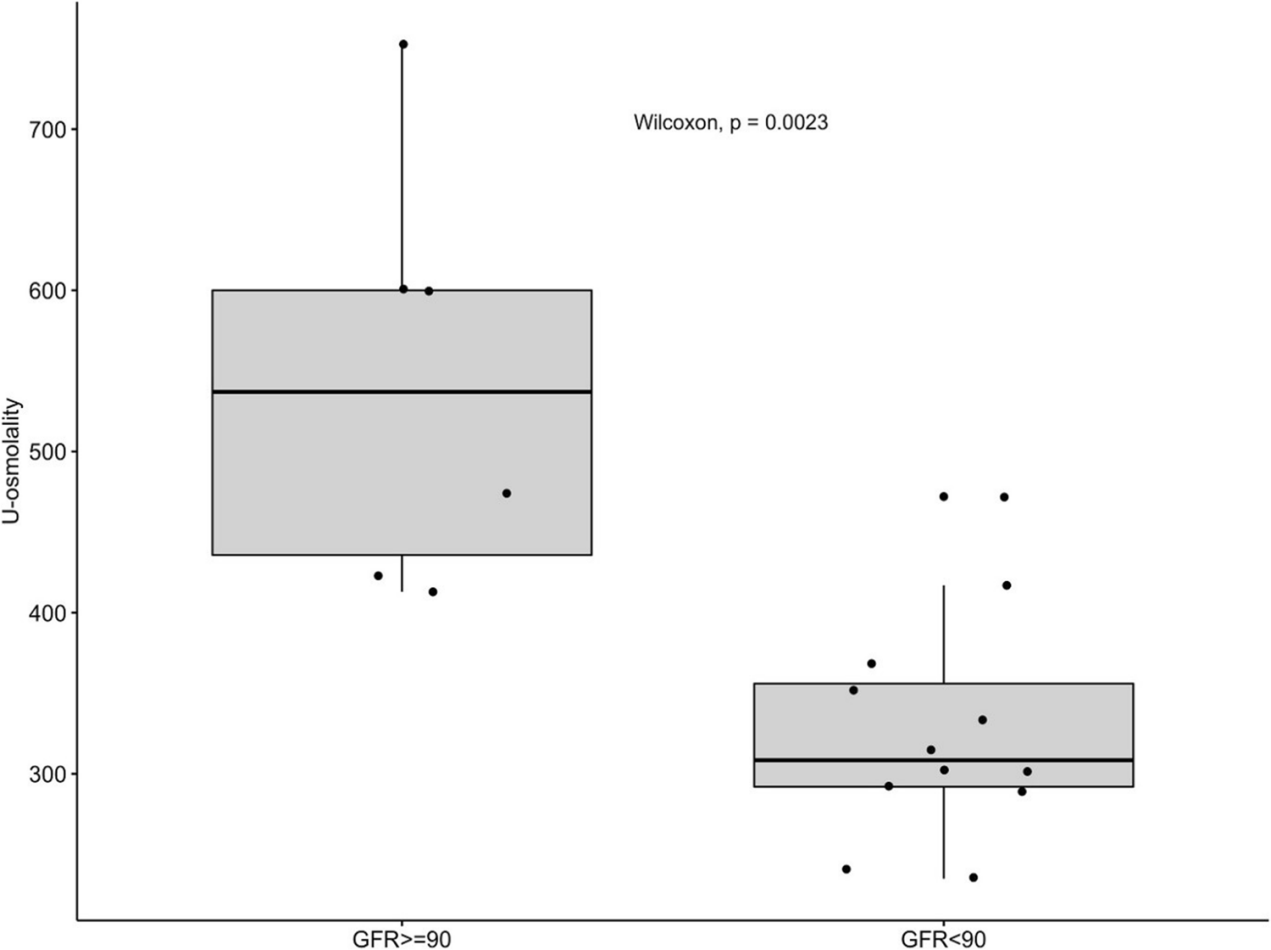
Kidney concentrating capacity in children with CKD1 and CDK stages 2–4

Twelve children (67%) were defined as having CKD stages 2–4 with decreased eGFR. The median concentrating capacity was significantly higher in children with normal GFR (CKD 1) when compared to children with CKD stages 2–4 (544 mOsmol/kg, IQR 484–602 vs. 327 mOsmol/kg, IQR 281–396, *p* = 0.002, Fig. 2).

The median concentrating capacity was not significantly different between children with early (< 1 year, *n* = 8) or late (> 1 year, *n* = 10) initial clinical presentation (397 mOsmol/kg, IQR 325–465 vs. 401 mOsmol/kg, IQR 384–564, *p* < 0.05).

Thirteen children (72%) were on antihypertensive drugs at the time of the study. Sixteen children (89%) were defined as hypertensive based on actual office BP levels or the use of antihypertensive drugs. The median systolic BPI was 0.99 (IQR 0.96–1.16) and diastolic BPI 0.96 (IQR 0.85–1.15); the median hypertension score was 2 (IQR 1–3). Eleven children (61%) were defined as hypertensive based on actual office BP levels, regardless of the use of antihypertensive drugs. The median concentrating capacity was significantly lower in children with hypertensive actual BP (BP index ≥ 95th percentile regardless of drugs) when compared to children with normotensive actual office BP (353 mOsmol/l, IQR 329–402 vs. 472 mOsmol/kg, IQR 282–546, *p* < 0.05).

**Fig. 3:**
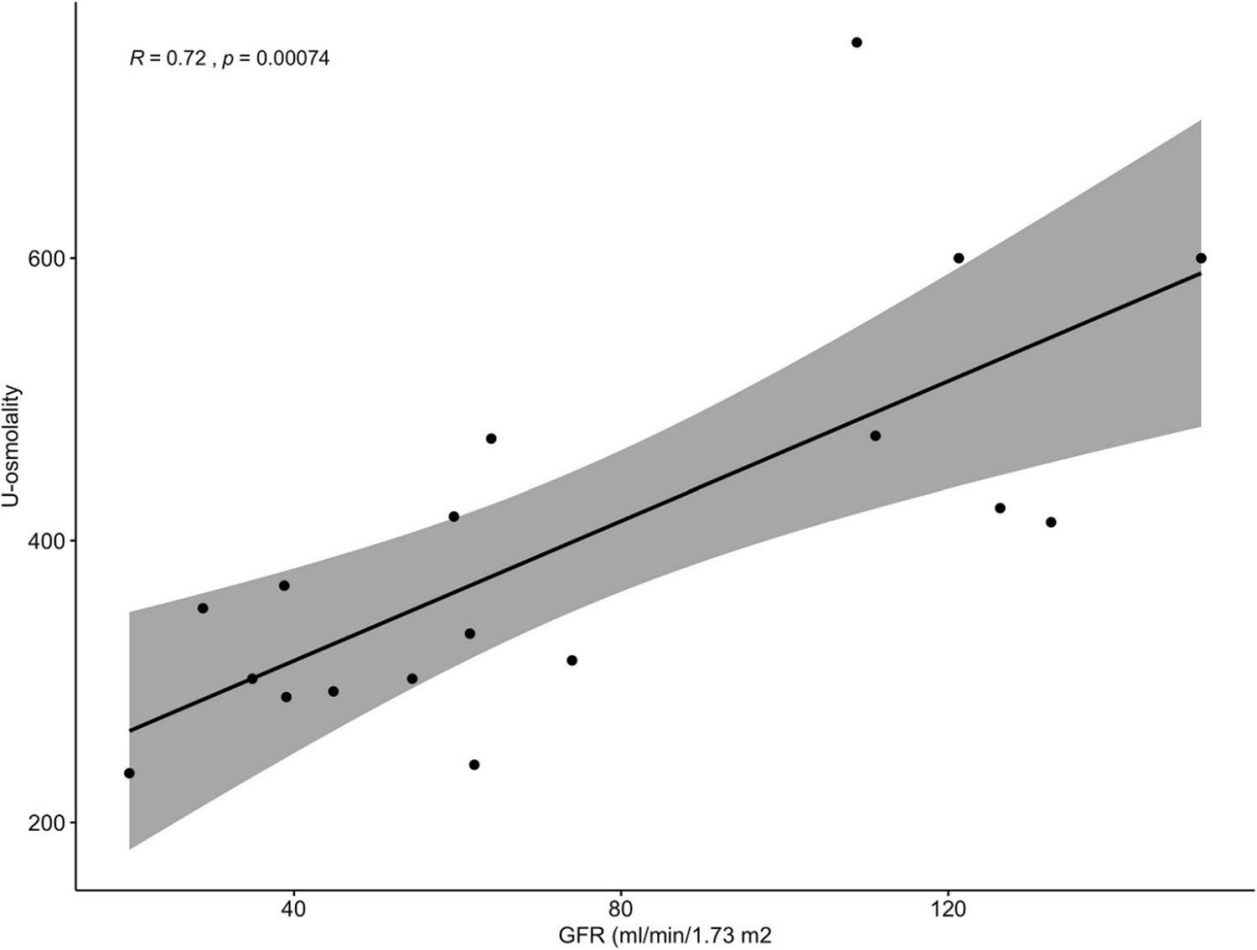
Correlation between kidney concentrating capacity and eGFR

**Fig. 4:**
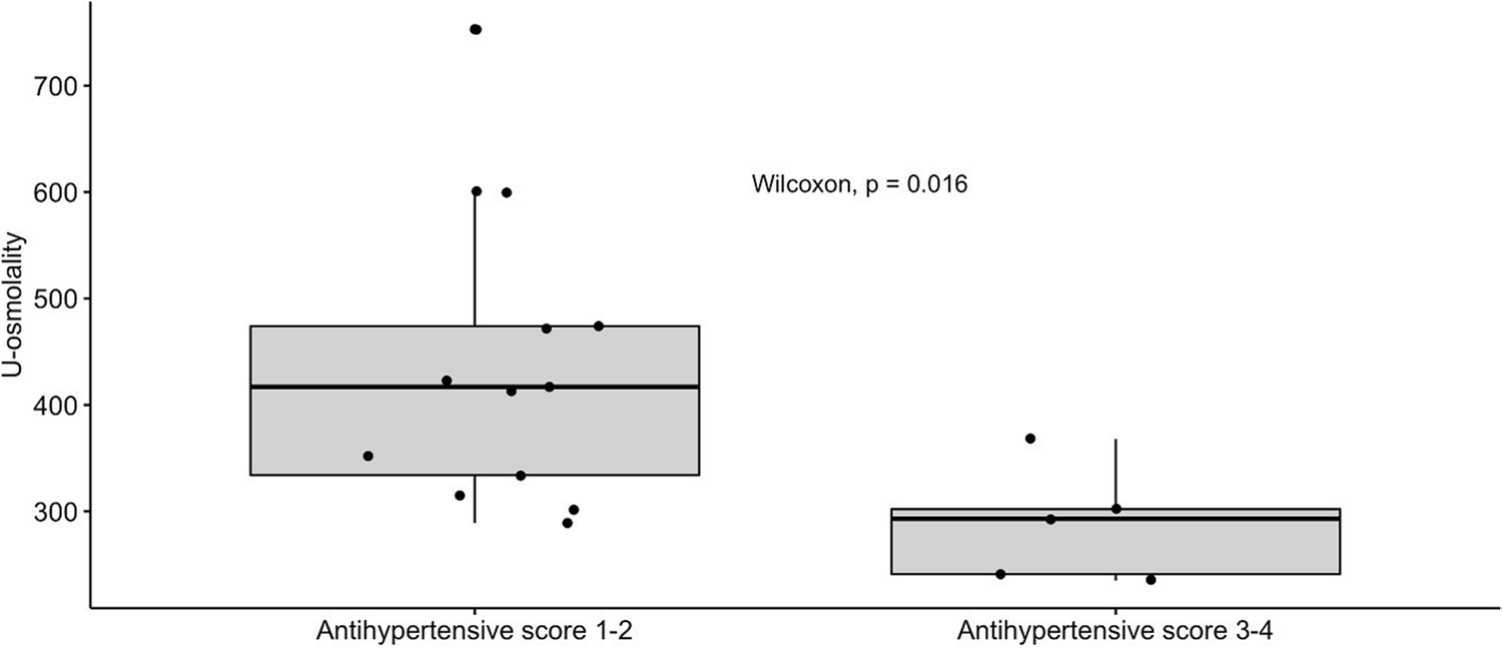
Kidney concentrating capacity in patients with different hypertension scores (hypertension score: 1 antihypertensive drug = 1 point, 2 drugs = 2 points etc.; hypertension by BP measurement regardless of drugs = 1 point)

Correlations between kidney concentrating capacity and different parameters are shown in Table 2. The correlation between concentrating capacity was significant for eGFR (Fig. 3) and hypertension score (Fig. 4), but not for kidney length, age, or proteinuria. The multivariate model showed eGFR as the independent predictor of kidney concentrating capacity (estimate standard error − 188.75, *p* = 0.01).

**Table 2:** Correlations between kidney concentrating capacity and clinical and laboratory parameters in children with ARPKD

	Correlation coefficient with urine osmolality (mOsmol/kg)	*p*−value
Age (years)	*r* = 0.19	NS
Kidney length (SDS)	*r* = 0.04	NS
eGFR (ml/min/1.73 m^2^)	*r* = 0.72	*p*=0.0007
Hypertension score (points)	*r* = −0.53	*p*=0.034
Systolic BP index	*r* = −0.33	NS
Diastolic BP index	*r* = −0.30	NS
Proteinuria (mg/mmol crea)	*r* = −0.41	NS
Age at 1st manifestation (years)	*r* = 0.03	NS
Time since 1st manifestation (years)	*r* = 0.29	NS

## Discussion

Autosomal recessive polycystic kidney disease is a very rare (1:20,000 live births) but severe multisystemic, typically early-onset disease. It can lead to perinatal mortality in 20–30% of newborns and neonatal survivors throughout childhood, with chronic kidney failure prevalence in approximately 50% of them [1, 2]. The most common clinical complication besides chronic kidney disease and congenital hepatic fibrosis is arterial hypertension; this affects about 90% of patients with ARPKD [10]. As it is primarily a tubular disorder (mainly collecting duct), different signs of tubulopathy such as impaired urinary dilution capacity associated with hyponatremia are present in 6–26% of children with ARPKD [2, 19].

In our previous study [6] along with further pediatric and adult studies [5, 7, 8, 20, 21], it has been shown that approximately 50% of patients with ADPKD have impaired kidney concentrating ability and reduced concentration capacity. These impairments were linked to ambulatory BP and the number of kidney cysts, but not to GFR. These findings have shown an association between kidney structural anomalies such as count of kidney cysts and functional anomalies of the tubuli such as impaired kidney concentrating ability.

Contrary to several studies regarding concentrating capacity in patients with ADPKD, to the best of our knowledge, there is only one small study on kidney concentrating capacity in patients with ARPKD. In this 1975 study, Anand et al. investigated urinary concentrating ability following vasopressin administration in five children with infantile polycystic kidney disease, an old term for ARPKD [9]. They demonstrated that the urinary concentrating ability was impaired in all five children (100%). They hypothesized that the inability to excrete maximally concentrated urine could be caused by anomalies in the tubular cells or changes in medulla due to increasing cysts.

In another clinical study on ARPKD, Kaplan et al. showed spontaneous urine osmolality in eight hyponatremic children and reported that it was inappropriately high at 119–220 mOsmol/kg [19].

In an animal model of a rat that phenotypically resembles ARPKD, Guay-Woodford et al. showed impaired urine-concentrating capacity [10]. At 3 weeks of age, the rats exhibited low urine osmolality after 2 h of fasting, along with elevated plasma creatinine and proteinuria, while showing parallel decreased glomerular filtration. Interestingly, urine osmolality was very similar (394 mOsmol/kg) to the urine osmolality in the children of our current study (389 mOsmol/kg) and three times lower than in unaffected animals (961 mOsmol/kg).

Our study is the second and largest study on kidney concentrating capacity of patients with ARPKD. It showed that 100% of ARPKD children have a decreased concentrating capacity; this impairment is thus more severe than in children with ADPKD (mean urine osmolality 389 mOsmol/kg in ARPKD vs. 882 mOsmol/kg in ADPKD) [6]. The significant correlation between concentrating capacity with eGFR is suggestive of an association between tubular and glomerular function in children with ARPKD, and eGFR was an independent predictor of kidney concentrating capacity. We could not find any similar correlation in patients with ADPKD in our previous study [6]. The reason for this difference in both polycystic kidney diseases could be the fact that all children with ADPKD had normal GFR and this thereby precludes any association due to the lack of children with decreased GFR. The difference can also be explained by the different affected nephron segments in ADPKD (all nephron segments) and ARPKD (predominant collecting duct where the final stage of urine concentration takes place). A correlation between concentrating capacity with GFR, similar to ARPKD, has also been demonstrated in non-genetic CKD, such as in patients with posterior urethral valves or reflux nephropathy [22, 23].

The children with ARPKD have a defect in both urine-diluting and urine-concentrating capacities [2, 9]. With disease progression, the microcystic lesions predominantly involve the cortical and medullary collecting ducts, causing further impairment of urine concentrating capacity.

The kidney concentrating capacity test has been performed in many other genetic and non-genetic CKD conditions, such as vesicoureteral reflux, posterior urethral valves, post-acute pyelonephritis, hypercalciuria, Bartter syndrome, Fanconi syndrome, tubulointerstitial nephritis, and post-hemolytic-uremic syndrome [22–29]. The tests showed impaired concentrating ability in a substantial proportion of children; therefore, it can be stated that a described concentration defect is not specific to ARPKD but is a nonspecific marker of distal tubular defect.

In our previous study on children with ADPKD, we found an association between ambulatory BP and kidney concentrating capacity [6]. In our current study, however, we did not find any similar association with office BP. As a possible explanation of the lack of correlation between BP and concentrating capacity in ARPKD, in comparison to children with ADPKD, we could ascertain that it might be the superior accuracy of 24 h ABPM regarding the reflection in the real BP of the patients. This may have been improved by including repeated office BP measures or home BP measurements rather than single measurements. Nevertheless, we have found a weak but significant correlation between concentrating capacity and hypertension score. This further exemplifies the heaviness of hypertension using the hypertensive score that also includes antihypertensive therapy rather than using only actual office BP, which can be decreased by the antihypertensives. Therefore, despite the lack of association between actual BP and kidney concentrating capacity, the association with hypertension continues to score further points. This might be explained by the fact that children with an elevated BP and hypertension have a more progressed state of ARPKD. In adults as well as pediatric patients with ADPKD, the concentrating capacity is linked to BP and hypertension [30, 31]. Contrary to the children with ARPKD, no correlation was found with actual BP. This could be related to the fact that the majority of children were treated with antihypertensive drugs, which preclude a correlation between tubular function and BP as in ADPKD children. Therefore, we used a hypertension score by Guidi et al. [17] to quantify the severity of hypertension more accurately, rather than solely considering the actual BP level.

Contrary to the children with ADPKD, we could not demonstrate any correlation between concentrating capacity and kidney length in ARPKD children. This could be due to the low accuracy and sensitivity of ultrasound derived kidney length or the fact that the relative kidney size (given as SDS) decreases relatively as the children are growing and the disease is progressing. Therefore, unlike in ADPKD, kidney size does not correlate with glomerular rates as well as tubular kidney functions in ARPKD [32].

It is very unlikely that the impaired concentrating capacity would be caused by antihypertensive therapy with diuretics, as only one patient received this drug that can influence the urinary concentrating capacity.

There are several possible pathophysiological mechanisms of impaired kidney concentrating capacity in ARPKD, such as structural alteration of the distal tubules and collecting ducts due to cyst formation, alterations in vasopressin receptor (V2R), ion channels such as epithelial sodium channels (ENaC) or aquaporins (AQPR) [33].

Impaired concentrating capacity that is present in children with ARPKD could have direct clinical relevance for patients, such as the increased risk of dehydration and/or a need for higher water intake or risk for enuresis.

There are several limitations to our study. Firstly, its retrospective design can produce a possible selection bias in the results. However, the kidney concentrating capacity test was performed in the majority of the children as part of the routine investigation of tubular function in children with tubular disorders such as ARPKD. Secondly, the small number of patients can hamper the statistical power. Therefore, our small retrospective study could not answer the question whether impaired concentrating capacity is truly an independent risk factor for progression of the disease. Future larger prospective studies would be beneficial and need to be performed in order to answer this question.

In conclusion, this second and largest study on kidney concentrating capacity in humans with ARPKD showed that it is decreased in most children with this disorder and is linked to glomerular filtration and hypertension but not to kidney size or proteinuria.

## Supplementary information

Below is the link to the electronic supplementary material.

Graphical Abstract (PPTX 90 KB)

## References

[1] Gimpel C, Liebau MC, Schaefer F (2020). Systematic review on outcomes used in clinical research on autosomal recessive polycystic kidney disease-are patient-centered outcomes our blind spot?. Pediatr Nephrol.

[2] Guay-Woodford LM, Bissler JJ, Braun MC, Bockenhauer D, Cadnapaphornchai MA, Dell KM, Kerecuk L, Liebau MC, Alonso-Peclet MH, Shneider B, Emre S, Heller T, Kamath BM, Murray KF, Moise K, Eichenwald EE, Evans J, Keller RL, Wilkins-Haug L, Bergmann C, Gunay-Aygun M, Hooper SR, Hardy KK, Hartung EA, Streisand R, Perrone R, Moxey-Mims M (2014). Consensus expert recommendations for the diagnosis and management of autosomal recessive polycystic kidney disease: report of an international conference. J Pediatr.

[3] Wilson PD (2004). Polycystic kidney disease. N Engl J Med.

[4] Fonck C, Chauveau D, Gagnadoux MF, Pirson Y, Grünfeld JP (2001). Autosomal recessive polycystic kidney disease in adulthood. Nephrol Dial Transplant.

[5] Kääriäinen H, Koskimies O, Norio R (1988). Dominant and recessive polycystic kidney disease in children: evaluation of clinical features and laboratory data. Pediatr Nephrol.

[6] Seeman T, Dusek J, Vondrák K, Bláhová K, Simková E, Kreisinger J, Dvorák P, Kyncl M, Hríbal Z, Janda J (2004). Renal concentrating capacity is linked to blood pressure in children with autosomal dominant polycystic kidney disease. Physiol Res.

[7] Gabow PA, Kaehny WD, Johnson AM, Duley IT, Manco-Johnson M, Lezotte DC, Schrier RW (1989). The clinical utility of renal concentrating capacity in polycystic kidney disease. Kidney Int.

[8] Zittema D, Casteleijn NF, Bakker SJ, Boesten LS, Duit AA, Franssen CF, Gaillard CA, Gansevoort RT (2017). Urine concentrating capacity, vasopressin and copeptin in ADPKD and IgA nephropathy patients with renal impairment. PLoS ONE.

[9] Anand SK, Chan JC, Liebermann E (1975). Polycystic disease and hepatic fibrosis in children: renal function studies. Am J Dis Child.

[10] Nauta J, Goedbloed MA, Herck HV, Hesselink DA, Visser P, Willemsen R, Dokkum RPEV, Wright CJ, Guay-Woodford LM (2000). New rat model that phenotypically resembles autosomal recessive polycystic kidney disease. J Am Soc Nephrol.

[11] Seeman T, Blažík R, Fencl F, Bláhová K, Obeidová L, Štekrová J, Weigel F, John-Kroegel U (2022). Ambulatory blood pressure and hypertension control in children with autosomal recessive polycystic kidney disease: clinical experience from two central European tertiary centres. J Hypertens.

[12] Janda J, Bláhová K, Marek V, Eliášek J (1988). Renal concentrating ability test in health children and adolescents. Kinderärztl Prax.

[13] Schwartz GJ, Brion LP, Spitzer A (1987). The use of plasma creatinine concentration to estimate glomerular filtration rate in infancy, childhood and adolescence. Pediatr Clin North Am.

[14] Schwartz GJ, Muñoz A, Schneider MF, Mak RH, Kaskel F, Warady BA, Furth SL (2009). New equations to estimate GFR in children with CKD. J Am Soc Nephrol.

[15] Kidney Disease: Improving Global Outcomes (KDIGO) CKD Work Group (2013). KDIGO 2012 clinical practice guideline for the evaluation and management of chronic kidney disease. Kidney Int Suppl.

[16] Lurbe E, Agabiti-Rosei E, Cruickshank JK, Dominiczak A, Erdine S, Hirth A, Invitti C, Litwin M, Mancia G, Pall D, Rascher W, Redon J, Schaefer F, Seeman T, Sinha M, Stabouli S, Webb NJ, Wühl E, Zanchetti A (2016). 2016 European Society of Hypertension guidelines for the management of high blood pressure in children and adolescents. J Hypertens.

[17] Guidi E, Bianchi G, Rivolta E, Ponticelli C, Quarto di Palo F, Minetti L, Polli E (1985). Hypertension in man with a kidney transplant: role of familial versus other factors. Nephron.

[18] Rosenbaum DM, Korngold E, Teele RL (1984). Sonographic assessment of renal length in normal children. Am J Roentgenol.

[19] Kaplan BS, Fay J, Shah V, Dillon MJ, Barratt TM (1989). Autosomal recessive polycystic kidney disease. Pediatr Nephrol.

[20] Marild S, Rembratt A, Jodal U, Norgaard JP (2001). Renal concentrating capacity test using desmopressin at bedtime. Pediatr Nephrol.

[21] Grantham JJ (2012). Bully renal cysts knock down urine-concentrating capacity in the early round. Clin J Am Soc Nephrol.

[22] Heikkilä J, Jahnukainen T, Holmberg C, Taskinen S (2021). Association of renal glomerular and tubular function with renal outcome in patients with posterior urethral valves. Urology.

[23] Dinneen MD, Duffy PG, Barratt TM, Ransley PG (1995). Persistent polyuria after posterior urethral valves. Br J Urol.

[24] García-Nieto V, García-Rodríguez VE, Luis-Yanes MI, Monge M, Arango-Sancho P, Garin EH (2019). Eur J Pediatr.

[25] García-Nieto V, González-Cerrato S, Luis-Yanes MI, Monge-Zamorano M, Reyes-Millán B (2014). Decreased concentrating capacity in children with febrile urinary tract infection and normal 99mTc-dimercaptosuccinic acid scan: does medullonephritis exist?. World J Pediatr.

[26] Kikuchi M, Sato M, Chiba A, Chiba Y, Nagao K, Suzuki T, Fujigaki Y, Hoshino H (1997). Studies on the site of renal tubular defect in Bartter’s syndrome. Acta Paediatr Jpn.

[27] Janda J, Rambousek V, Kolský A, Stejskal J, Klimesová D, Feber J (1990). Acute interstitial nephritis with uveitis in children and adolescents. Cesk Pediatr.

[28] Rossi R, Helmchen U, Schellong G (1992). Tubular function and histological findings in ifosfamide-induced renal Fanconi syndrome–a report of two cases. Eur J Pediatr.

[29] de Jong M, Monnens L (1988). Haemolytic-uraemic syndrome: a 10-year follow-up study of 73 patients. Nephrol Dial Transplant.

[30] Gabow PA, Chapman AB, Johnson AM, Tangel DJ, Duley IT, Kaehny WD (1990). Renal structure and hypertension in autosomal dominant polycystic kidney disease. Kidney Int.

[31] Seeman T, Dusek J, Vondrichová H, Kyncl M, John U, Misselwitz J, Janda J (2003). Ambulatory blood pressure correlates with renal volume and number of renal cysts in children with autosomal dominant polycystic kidney disease. Blood Press Monit.

[32] Gunay-Aygun M, Avner ED, Bacallao RL, Choyke PL, Flynn JT, Germino GG, Guay-Woodford L, Harris P, Heller T, Ingelfinger J, Kaskel F, Kleta R, LaRusso NF, Mohan P, Pazour GJ, Shneider BL, Torres VE, Wilson P, Zak C, Zhou J, Gahl WA (2006). Autosomal recessive polycystic kidney disease and congenital hepatic fibrosis: summary statement of a first National Institutes of Health/Office of Rare Diseases conference. J Pediatr.

[33] Sudarikova AV, Vasileva VY, Sultanova RF, Ilatovskaya DV (2021). Recent advances in understanding ion transport mechanisms in polycystic kidney disease. Clin Sci.

