# Supplementary material for: Kidney concentrating capacity in children with autosomal recessive polycystic kidney disease is linked to glomerular filtration and hypertension
Source: Pediatr Nephrol. 2022 Dec 20;38(7):2093–100. doi: 10.1007/s00467-022-05834-5 (PMC10234879; doi:10.1007/s00467-022-05834-5)
Supplement: Supplementary file 1 — Graphical Abstract (PPTX 90 KB) [file 467_2022_5834_MOESM1_ESM.pptx]

## Slide 1
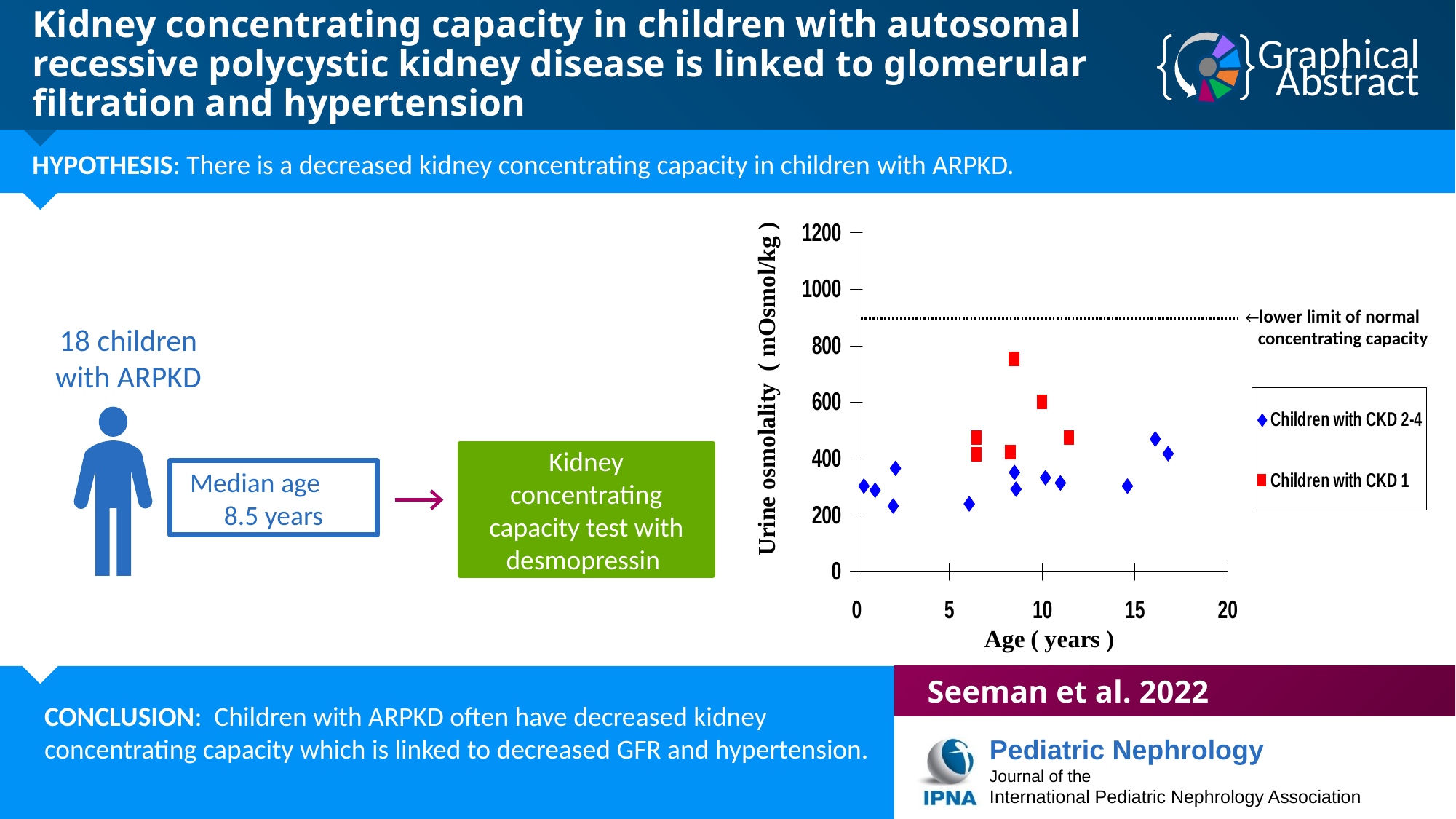

Kidney concentrating capacity in children with autosomal recessive polycystic kidney disease is linked to glomerular filtration and hypertension
HYPOTHESIS: There is a decreased kidney concentrating capacity in children with ARPKD.
←lower limit of normal
 concentrating capacity
18 children with ARPKD
Kidney concentrating capacity test with desmopressin
Median age 8.5 years
Seeman et al. 2022
CONCLUSION: Children with ARPKD often have decreased kidney concentrating capacity which is linked to decreased GFR and hypertension.
